# Massive B-Cell Infiltration and Organization Into Artery Tertiary Lymphoid Organs in the Aorta of Large Vessel Giant Cell Arteritis

**DOI:** 10.3389/fimmu.2019.00083

**Published:** 2019-01-29

**Authors:** Jacoba C. Graver, Annemieke M. H. Boots, Erlin A. Haacke, Arjan Diepstra, Elisabeth Brouwer, Maria Sandovici

**Affiliations:** ^1^Department Rheumatology and Clinical Immunology, University Medical Center Groningen, University of Groningen, Groningen, Netherlands; ^2^Department of Pathology and Medical Biology, University Medical Center Groningen, University of Groningen, Groningen, Netherlands

**Keywords:** giant cell arteritis, B-cell, tertiary lymphoid organs, aorta, plasma cells

## Abstract

Giant cell arteritis (GCA) can be classified into Cranial(C)-GCA and Large Vessel(LV)-GCA. Based on analysis of temporal arteries, GCA is postulated to be T-cell-mediated. Recently, a disturbed B-cell homeostasis was documented in newly diagnosed GCA patients. In the current study, we assessed the presence of B-cells and their level of ectopic organization in the aorta of LV-GCA patients. Aorta tissue samples of 9 histologically-proven LV-GCA patients and 22 age- and sex-matched atherosclerosis patients who underwent aortic aneurysm surgery were studied by immunohistochemistry. Sections were stained for B-cells, T-cells, follicular dendritic cells, high endothelial venules, germinal center B-cells, proliferating B-cells, macrophages, and plasma cells. Aortas of LV-GCA patients showed massive infiltration of B-cells, which clearly outnumbered T-cells, as opposed to C-GCA patients where, as previously reported, T-cells outnumber B-cells. B-cells were mainly found in the adventitia of the vessel wall and were organized into artery tertiary lymphoid organs. These tertiary lymphoid organs had germinal centers, proliferating B-cells and plasma cell niches. In conclusion, we found massive and organized B-cell infiltrates in the aorta of LV-GCA patients, which is in line with the previously documented decrease of circulating B-cells in active GCA. Our data indicate a role for B-cells in the pathogenesis of GCA and thus evoke further investigation into the factors determining the tissue tropism and organization of B-cells in GCA.

## Introduction

Giant cell arteritis (GCA) can be classified into Cranial(C)-GCA and Large Vessel(LV)-GCA. Even with modern imaging techniques, diagnosing LV-GCA remains difficult due to its non-specific clinical symptoms, such as weight loss or fever. LV-GCA patients can therefore present with late stage complications such as aortic aneurysm or dissection (1). Knowledge about the immunopathogenesis of GCA is almost exclusively based on the analysis of temporal artery biopsies (TAB) in C-GCA. GCA is therefore regarded as a T-cell mediated disease and it is assumed that the immunopathogenesis at the level of the aorta is similar to the processes in the temporal artery (2). B-cells are underexplored in GCA due to the lack of disease specific autoantibodies and the low number of B-cells in TAB (3, 4). However, recent findings do suggest a role for B-cells in GCA. During active GCA, peripheral B-cell counts were reduced and their numbers correlated inversely with markers of systemic inflammation (5). During treatment, B-cell counts normalized, suggesting that B-cells are redistributed to the tissues during active disease and return to the circulation during remission. These returning effector B-cells demonstrated an enhanced capacity to produce the pro-inflammatory cytokine Interleukin(IL)-6. Furthermore, B-cells were able to organize into artery tertiary lymphoid organs (ATLOs) in TAB from C-GCA patients (6, 7). ATLOs were also documented in other vasculitides that can affect the aorta, like Takayasu Arteritis (8) as well as in atherosclerosis (9). The data indicated that B-cells are more involved in the pathology of GCA than previously believed and urged this histopathological study of LV-GCA aorta.

## Materials and Methods

### Study Population

In this retrospective study, aorta tissue samples of 9 histologically-proven LV-GCA patients and 22 age- and sex-matched atherosclerosis patients who underwent aortic aneurysm surgery were studied. Patients' clinical and laboratory data at time of surgery were extracted from medical records. All procedures were in compliance with the Declaration of Helsinki. Consent from the Internal Review Board and written patient consent were not required under the Dutch law for human medical research (WMO) since this is a retrospective, non-interventional study. Patients were informed that obtained medical data could be used for research purposes in accordance with privacy rules. Number Research Register UMCG: 201800370.

### Histopathology and Immunohistochemistry

Aorta tissue was formalin fixed, paraffin embedded and sectioned at 3 μm thickness. Every first section was stained for Hematoxylin & Eosin (H&E). Consecutive sections were deparaffinized in xylene and rehydrated with graded ethanol. Antigen retrieval was performed and followed by endogenous peroxidase blockade. Staining with antibodies detecting CD20 (B-cells), CD3 (T-cells), CD21 [follicular dendritic cells (FDC)], Peripheral node Addressin [PNAd, high endothelial venules (HEV)], CD68 (macrophages), CD138 (plasma cells) was performed. See Supplementary Material (Table S1) for all antibodies and antigen retrieval methods used. Sections were incubated with primary anti-human antibodies for 60 min at room temperature. Next, sections were incubated with secondary antibodies for 30 min. Secondary antibodies used were rabbit anti-mouse HRP (DAKO P0260, 1:50), rabbit anti-rat HRP (DAKO P0450, 1:50) and rabbit anti-mouse UltraVision One detection system HRP polymer (Thermo Fisher Scientific TL-125-PHJ). After incubation sections were treated with DAB for visualization and counterstained with hematoxylin.

Staining for B-cell lymphoma six protein (Bcl6, germinal center B-cells), adipophilin (atherosclerosis, foam cells), IgM, IgG, and IgG4 was performed on a Ventana Benchmark platform. Appropriate negative and positive controls were used. All stained tissue sections were scanned using a Nanozoomer Digital Pathology Scanner (NDP Scan U 10074-01, Hamamatsu Photonics K.K.) before being analyzed.

### Immunofluorescence for Detection of Proliferating B Cells

Double-labeling immunofluorescence staining was performed on formalin-fixed, paraffin-embedded aortic tissue. The tissue was deparaffinized followed by antigen retrieval. Blocking was performed with goat serum and avidin/biotin. Primary antibodies against CD20 and Ki-67 (Table S1) were incubated for 60 min followed by 30 min incubation of TRITC labeled IgG2A (1080-03, Southern Biotech) and FITC labeled IgG1A (1070-02, Southern Biotech) secondary antibodies, respectively. DAPI was used to stain nuclei. Images were taken with Leica DFC345 FX.

### Analysis of Tissue Staining

Analysis (blinded for diagnosis) was performed by 2 independent investigators. Semi-quantitative scoring of CD20 staining was performed using a 5-point scale (0 = no positive cells, 1 = occasional positive cells, 2 = moderate number of positive cells, 3 = large number of positive cells, presence of clusters, 4 = very large number of positive cells). To allow comparison of CD20 and CD3, the positivity ratio (positive pixels/total number of pixels) was assessed using Aperio ImageScope software (v12.1.0.5029, Aperio Technologies) on three areas representative of the entire tissue, selected based on the H&E staining. ATLO scoring was performed on tissues with a B-cell score of ≥3. A lymphocytic focus was scored as ATLO when it showed co-localized clusters of B-cells and T-cells that displayed a FDC network. Furthermore, macrophage staining was taken into account to distinguish granulomas (CD68^+^) from ATLOs (CD68-). ATLOs were analyzed for presence of germinal centers and HEV. Plasma cells per ATLO were scored on a 3-point scale (0 = no plasma cells, 1 = <10 plasma cells, 2 = >10 plasma cells). Atherosclerosis severity was determined by examining media damage at the atheromatous plaque. A 5-point scale was used with 0 = no atherosclerosis, 1 = very mild (intima hyperplasia with foam cells, media unaffected), 2 = mild (less than half of media affected by atherosclerosis), 3 = moderate (more than half of media affected by atherosclerosis), 4 = severe (media completely destructed by atherosclerosis). In addition, the number of plaques was documented.

### Statistical Analysis

Statistical analysis was performed with the Mann-Whitney-*U*-test or Fisher's exacts test. The Wilcoxon signed rank test was used for paired samples. Correlations were analyzed with Spearman's rank correlation coefficient. Analysis was performed with GraphPad Prism 7.0 and *p* < 0.05 (2-tailed) were considered significant.

## Results

### Patient Characteristics

**Figure 1:**
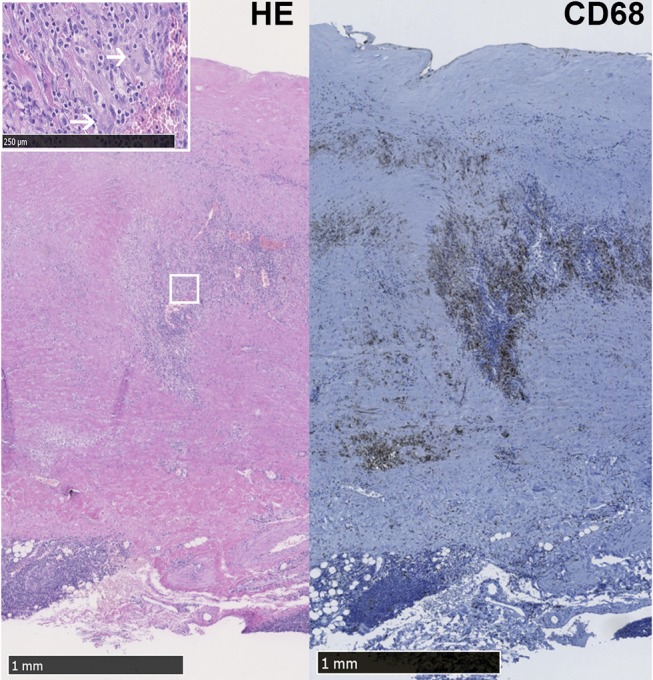
LV-GCA aorta with granulomatous inflammation and giant cells. Representative image of Hematoxylin Eosin (HE) and CD68+ macrophages in the media layer of the aorta from a LV-GCA patient. The white box shows magnified giant cells (white arrows) in the HE staining.

In the selected group of patients who presented with an aneurysm of the aorta, diagnosis of LV-GCA was based on histopathology. All LV-GCA patients showed granulomatous inflammation in the media and all but one contained giant-cells (Figure 1). None of the patients received glucocorticoids or other immunosuppressive treatment at the time of surgery. Two patients had chronic fatigue and one had night sweats at the time of surgery. However, no suspicion of GCA was raised by the cardiologist or cardio-thoracic surgeon before the surgery of the aorta aneurysm. After the histopathological examination of the aortic specimen, either an internist or rheumatologist was consulted in 7 out of 9 patients. Two patients died of complications after surgery. One patient received prednisolone treatment for 6 weeks after surgery. The other LV-GCA patients were not treated with glucocorticoids due to lack of clinical signs or symptoms of active GCA, as assessed by signs and symptoms of cranial GCA, measurement of the CRP, and/or ESR, evaluation of a blood pressure difference between the right/left brachial artery and/or femoral artery or 18F-fluorodeoxyglucose-positron emission tomography (FDG-PET) scan (4 out of 9 patients).

Four of the LV-GCA patients had a suspicion of C-GCA mentioned in their past history (5–11 years before aortic surgery). A TAB was performed in three of these patients and was positive in one patient. Of these 4 patients, one improved spontaneously (e.g., without glucocorticoid treatment) and 3 received prednisolone treatment at diagnosis. Unfortunately, the precise treatment duration could not be established retrospectively. The C-GCA patient with the positive TAB mentioned persistent fatigue after cessation of prednisolone treatment for C-GCA.

As a control, aorta tissue of age and sex matched atherosclerosis patients who presented with an aneurysm was taken into account. Detailed patient characteristics are shown in Table 1.

**Table 1:** Patient characteristics of LV-GCA and atherosclerosis groups.

	**LV-GCA**	**Atherosclerosis**
No. of patients	9	22
Age (years): mean (*SD*)	67.4 (8.8)	67.6 (7.0)
Gender, no. female (%)	6 (66.7)	14 (63.6)
No. of subjects on treatment with glucocorticoids/immunosuppression	0	0
No. of subjects with C-GCA in the medical history	4 suspected, 1 TAB proven (5–11 years before aortic surgery)	0
ESR (mm/h): median (IQR)^*^	12.5 (3.5–27.3)	15 (4–20)
CRP (mg/L): median (IQR)^#^	9 (5–11)	5 (5–11)
Total length of studied aortic tissue (mm): median (IQR)	64.7 (43.8–74)	54.1 (46.1–63.4)

*Reported data is from the time of aorta surgery*.

* *ESR; n = 6 for LV-GCA and n = 19 for atherosclerosis*.

# *CRP; n = 7 for LV-GCA and n = 19 for atherosclerosis. There was no significant difference between the groups regarding age, gender, ESR, CRP, and the total length of studied aortic tissue*.

### B-Cells Accumulate in the Aorta of LV-GCA Patients and Outnumber T-Cells

**Figure 2:**
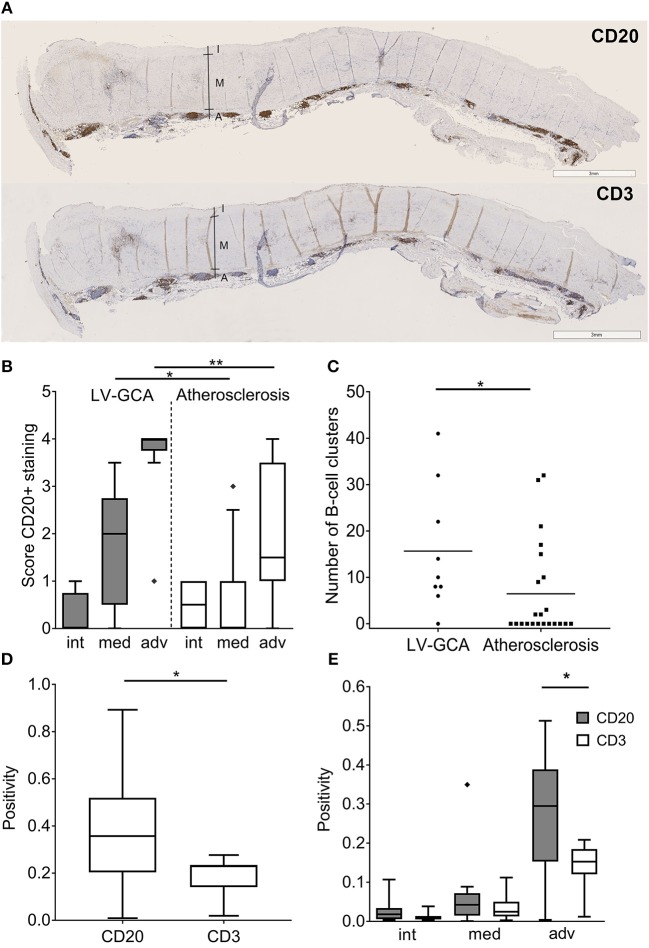
B-cells outnumber T-cells in aorta tissue from LV-GCA patients. **(A)** Representative images of CD20+ B-cells (upper panel) and CD3+ T-cells (lower panel) in the aorta of LV-GCA patients. I, intima; M, media; A, adventitia. **(B)** Semi-quantitative analysis of CD20+ B-cell abundance in aorta of LV-GCA and atherosclerosis patients for the intima (int), media (med), and adventitia (adv). Scoring was performed on a 5-point scale with 0 = no positive cells, 1 = occasional positive cells, 2 = moderate number of positive cells, 3 = large number of positive cells (clusters), 4 = very large number of positive cells. **(C)** The number of B-cell clusters (semi-quantitative score ≥3) in the tissue per patient. The line depicts the median. **(D)** Overall CD20 and CD3 positivity for the aorta, as quantified by pixel count of tree representative areas per tissue (*n* = 9). **(E)** CD20 and CD3 expression for the intima, media and adventitia of the aorta as quantified by pixel count. Three representative areas per tissue (*n* = 9) were analyzed. In the box and whisker plots (Tukey), boxes indicate median values and interquartile ranges. The Mann-Whitney *U*-test was used to compare both groups in **(B,C)**, the Wilcoxon signed rank test was used in **(D,E)**. ^*^P < 0.05, ^**^P < 0.01.

Massive infiltrates of B-cells were observed in the aorta of LV-GCA patients (Figure 2A). Most B-cells were observed in the adventitial layer. Two patterns of infiltrating B-cells were distinguished, an unorganized diffuse pattern and an organized clustered pattern. There were significantly more B-cells in the media and adventitia of LV-GCA patients compared to atherosclerosis patients (Figure 2B). LV-GCA patients had significantly more B-cell clusters per patient (Figure 2C). While T-cells clearly outnumber B cells in temporal artery tissue, B-cells clearly outnumbered T-cells in the aorta of LV-GCA patients (Figure 2D). There were significantly more B-cells compared to T-cells in the adventitia (Figure 2E).

### B-Cells in the Aorta of LV-GCA Patients Organize Into ATLOs

**Figure 3:**
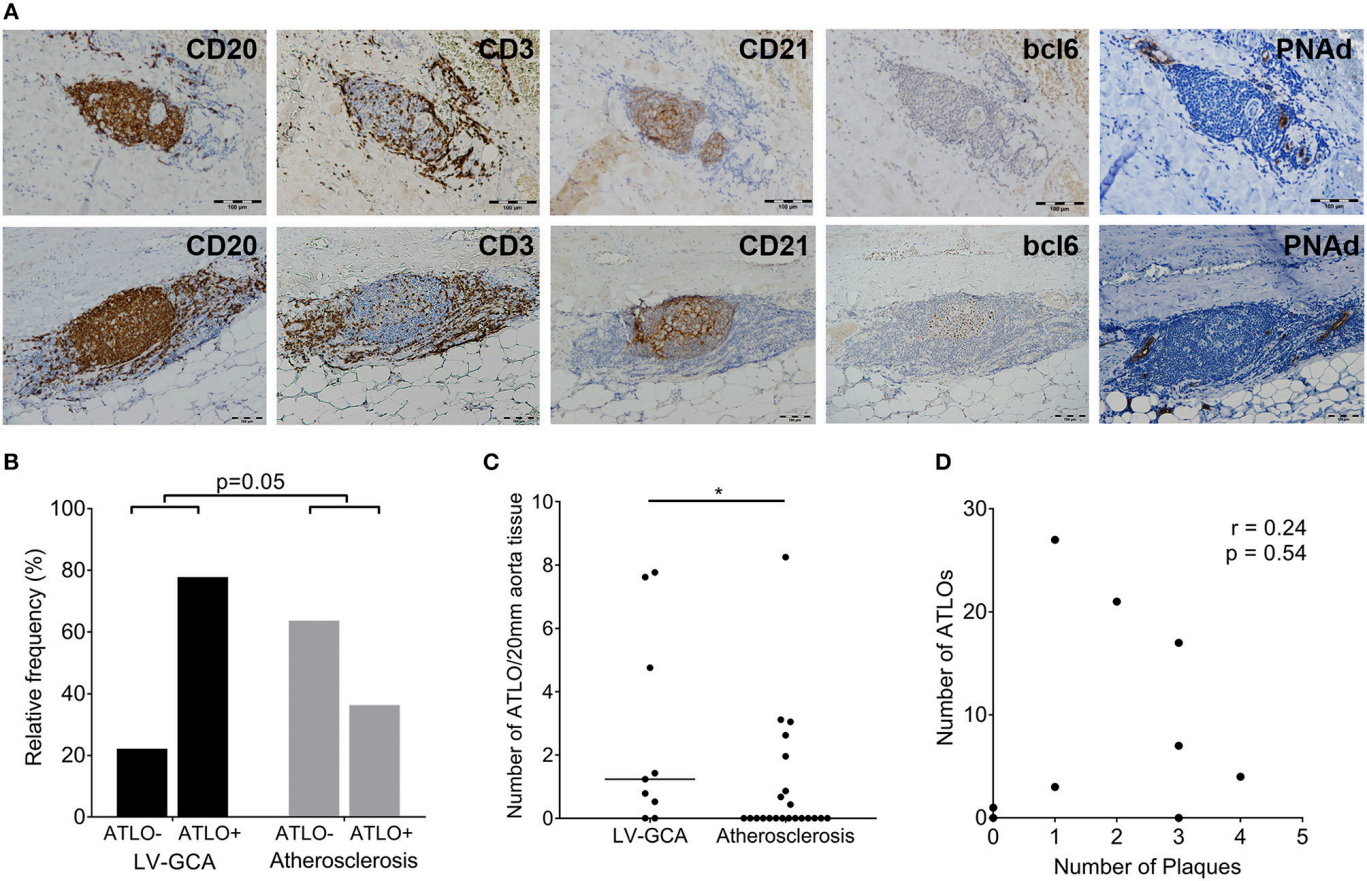
ATLOs in the aorta of LV-GCA and atherosclerosis patients. **(A)** Representative images of consecutive sections of aorta tissue showing in brown B-cells (CD20), T-cells (CD3), FDC (CD21), GCs (bcl6), and HEV (PNAd). The upper panel shows an ATLO without GC (bcl6–) and the lower panel an ATLO with GC (bcl6+). **(B)** Relative frequency of patients with ATLOs (ATLO+) and without ATLOs (ATLO–) in the aorta for both LV-GCA and atherosclerosis patients. **(C)** Number of ATLOs per 20 mm of aortic tissue for both LV-GCA and atherosclerosis patients. **(D)** Lack of association between atherosclerosis (number of plaques) and the number of ATLOs for LV-GCA patients. Fischer's exact test was used in **(B)**, Mann-whitney *U*-test in **(C)** and Spearmans *r* was used for **(D)**. ^*^P < 0.05.

**Figure 4:**
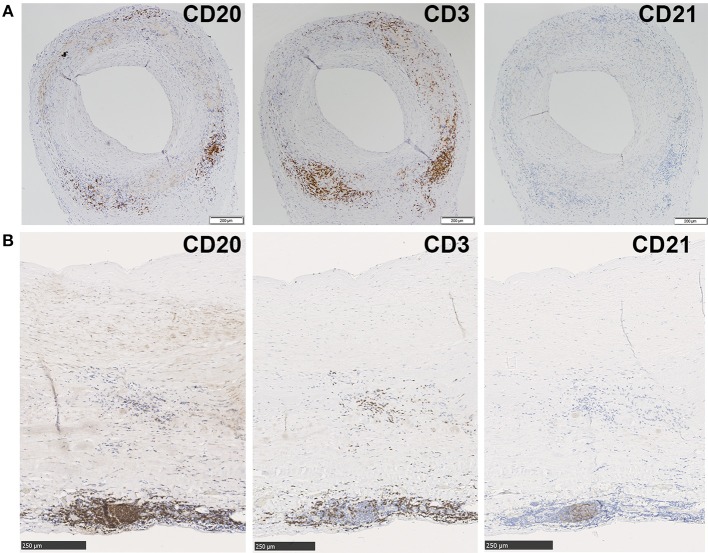
B-cell, T-cell and FDC network staining in TAB and aorta tissue from one patient who experienced C-GCA 5 years before aneurysm surgery. Immunohistochemical stainings show from left to right, CD20+ B-cells, CD3+ T-cells, and no CD21+ FDC network in consecutive sections of a GCA positive TAB **(A)** and CD20+ B-cells, CD3+ T-cells and a clear CD21+ FDC network in consecutive sections of GCA positive aorta **(B)**.

Aortas were further assessed for organization into ATLOs (Figure 3A). ATLOs were present in 77.8% of LV-GCA tissues as opposed to 36.4% of atherosclerosis tissues (Figure 3B). All ATLOs were located in the adventitial layer, close to the media. In LV-GCA patients, ATLOs were localized at a level corresponding to a granuloma in the media in 53% of cases. We also documented the presence of germinal centers in ATLOs in 66.7% of LV-GCA aortas compared to 27.3% of atherosclerosis aortas (*p* = 0.06). All tissues containing ATLOs showed PNAd+ HEV at the border of T-cell rich areas (Figure 3A). The number of ATLOs per patient was significantly higher in LV-GCA (Figure 3C). Furthermore, the number of ATLOs containing a germinal center was also significantly higher in LV-GCA aortas (*p* = 0.02). To exclude that observed differences in ATLO presence were associated with atherosclerosis instead of GCA, atherosclerosis severity was scored in all patients. We found no significant differences between the LV-GCA and atherosclerosis group regarding atherosclerosis severity. Percentages of atherosclerosis severity score 1, 2, 3, and 4 were, respectively: 33, 33, 22, and 11% for the LV-GCA group and 23, 36, 18, and 23% for the atherosclerosis group. Furthermore, no association was found between the number of atherosclerotic lesions and the number of ATLOs from LV-GCA patients (Figure 3D). To determine if B-cells and ATLOs were also present in the positive TAB from the LV-GCA patient that experienced C-GCA 5 years before aortic surgery, we stained the TAB for B-cells, T-cells, and FDC networks (Figure 4A). B-cells were presented on three different areas of the adventitia/media border. These B-cells were clustered in one location and unorganized diffuse in the other two locations. T-cells were abundantly present at the same areas as the B-cells. FDC networks could not be detected inside the TAB. This data indicate a degree of T/B cell organization in the TAB of this patient, however not (yet) as ATLO. This patient did show ATLO formation in the adventitia of the aorta (Figure 4B).

### Plasma Cells and Proliferating B-Cells Are Present in the ATLOs of LV-GCA Patients

**Figure 5:**
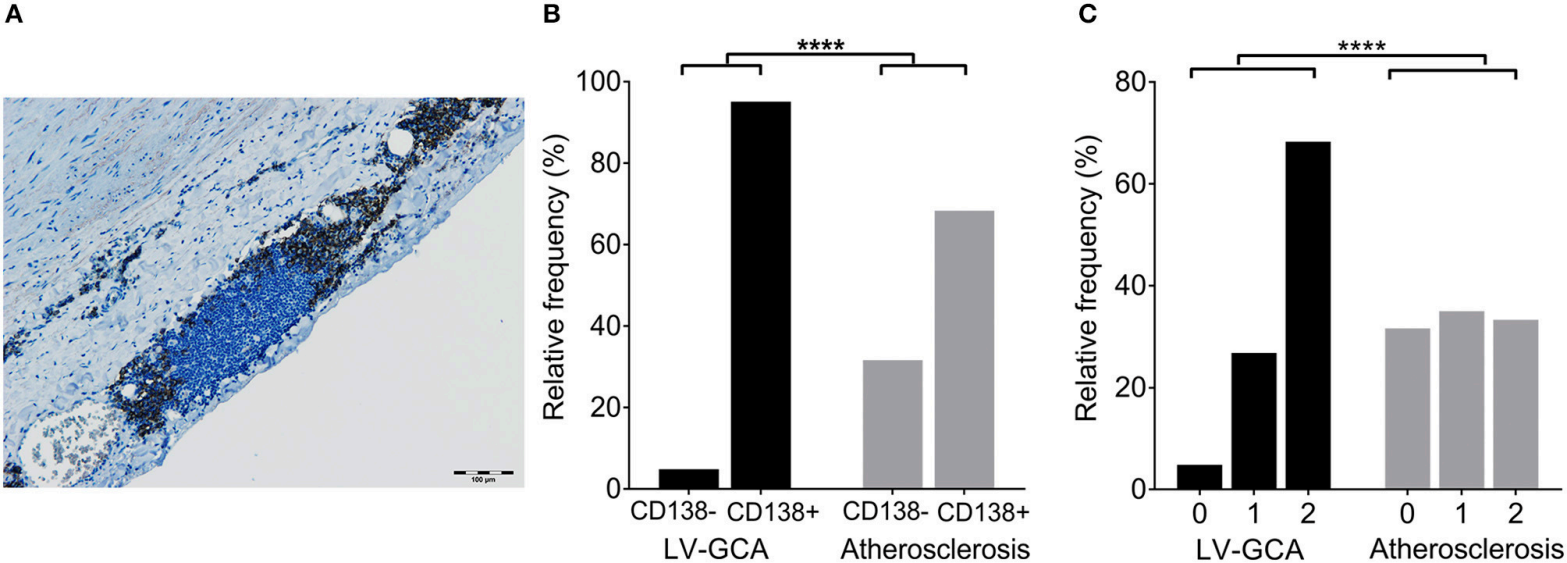
Plasma cells in ATLOs of LV-GCA and atherosclerosis patients. **(A)** Representative image of an ATLO with CD138+ plasma cells in niches (brown). **(B)** Histogram showing the frequency of plasma cell niches per ATLO. CD138-: no plasma cells present, CD138+: plasma cells present. **(C)** Histogram showing the frequency distribution of plasma cell score. Plasma cells were scored semi-quantitatively by 2 independent investigators on a 3-point scale (0–2) with 0 = no positive cells, 1 = few positive cells (< 10), and 2 = moderate to large number of positive cells (>10). Fischer's exact test was used in **(B)** and the Mann-Whitney *U*-test was used in **(C)**. ^****^P < 0.0001.

**Figure 6:**
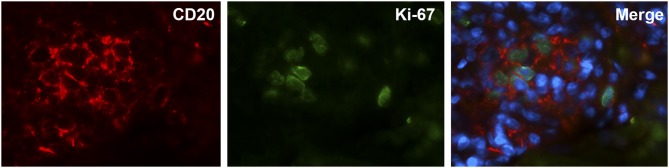
Locally proliferating B-cells in ATLOs of LV-GCA patient. Immunofluorescence staining showing from left to right: single staining for B-cells (CD20, red), single staining for proliferation marker Ki-67 (green), double staining for B-cells, and proliferation maker Ki-67 together with DAPI staining of nuclei (blue).

Staining with CD138 was performed to assess the presence of plasma cell niches which is a feature of ATLOs (Figure 5A). There were strikingly more ATLOs containing plasma cell niches in LV-GCA than in atherosclerosis tissues (Figure 5B). In addition, ATLOs from LV-GCA had a higher number of plasma cells in the niche compared to atherosclerosis tissues (Figure 5C). Most plasma cells expressed IgG and only a minority was positive for IgM. Only a small number of the IgG+ plasma cells expressed IgG4 (data not shown), excluding an IgG4-related aortitis. To assess if B-cells within the ATLOs are proliferating, double staining of CD20 and proliferation marker Ki-67 was performed and showed clear co-localization of these markers (Figure 6). In LV-GCA, Ki-67+ cells were also observed in B-cell rich areas that did not qualify as ATLO and in ATLOs without a germinal center.

## Discussion

Our data show massive infiltration of B-cells in the aorta of LV-GCA patients presenting with an aneurysm of the aorta. In the majority of patients, these B-cell infiltrates were organized into ATLOs with germinal centers and plasma cell niches. So far, knowledge of the inflammatory infiltrate in GCA has largely been based on temporal artery biopsies (TAB). However, in contrast to C-GCA, we found a dominance of B-cells over T-cells in the aorta of LV-GCA. These data suggest a preferential migration of B cells to the aorta (i.e., large arteries) instead of the temporal (i.e., cranial) arteries, which may be related to a different chemokine and chemokine receptor expression in the aorta as compared to the cranial arteries. The massive presence of B-cells in the aorta strongly support a role for B-cells in the pathogenesis of LV-GCA.

In the majority of LV-GCA aortas (78%) B-cells were organized into ATLOs. GCA is a disease of the elderly and thus coexistence with atherosclerosis is expected. While the presence of ATLO in atherosclerosis is well-known, (9) we demonstrated that our findings were not dependent on atherosclerosis. We observed a varying degree of organization in the aortic wall of LV-GCA patients; ranging from B- and T-cell aggregates to highly organized ATLOs containing a FDC network, HEV and germinal centers. This is in line with previously reported developmental stages of tertiary lymphoid organs (TLO) (10). All ATLOs were located in the adventitia, mostly at the border with the media. In the positive TAB of the patient that experienced C-GCA in the past we found B- and T-cell aggregates in the adventitia. However, no ATLO was found in this TAB and T-cells were more abundant than B-cells.

In atherosclerosis, ATLOs are reported to develop at the adventitial site opposite to the plaque (11). Remarkably, approximately half of the ATLOs in LV-GCA were located adjacent to a granuloma. Granulomas were found in all LV-GCA tissues. Interestingly, in aorta tissues from Takayasu Arteritis, only 29% of the patients displayed granulomas while ATLOs were found in 85% (8). The high abundance of granulomas in the aortic wall of LV-GCA patients reflects ongoing silent vascular inflammation, despite the lack of clinical symptoms. This observation is in line with the findings of Maleszewski et al. showing persistent inflammation in a second TAB at 12 months in 44% of GCA patients, despite treatment-induced clinical and biochemical remission (12). Ongoing silent vasculitis rather than early damage may be responsible for the development of aneurysm and dissection in LV-GCA.

Remarkably, most of the ATLOs in GCA patients contained a germinal center with proliferating B-cells. In secondary lymphoid organs, germinal centers are sites of class switching, affinity maturation, and B-cell differentiation (9, 13). The adventitial ATLOs of LV-GCA patients contained proliferating B-cells indicating local activity of B-cells within the germinal centers. The LV-GCA ATLOs also consistently contained plasma cells (95%) as opposed to ATLOs in atherosclerotic aortas (68%). The presence of active germinal centers and associated plasma cell niches suggest that humoral immune mechanisms are involved in LV-GCA. Auto antibodies related to the inflammatory response (e.g., not disease specific), including anti-ferritin and anti-endothelial cell antibodies have been documented in GCA (14–17), however, no disease-specific autoantibodies have been found so far.

Alternatively, B-cells may be involved in an autoantibody-independent manner. IL-6, a prominent cytokine in GCA, is produced with enhanced capacity by effector B-cells returning to the circulation after glucocorticoid-induced remission (5). Emerging data indicate that B-cell cytokines can modulate T-cell responses (18) and B-cells have been shown to augment T-cell-mediated autoimmune responses by IL-6 secretion (19). B-cells could thus contribute to the pro-inflammatory environment and help shape T-cell responses.

Our findings of organized B-cell architecture in LV-GCA may be related to disease duration. In chronic inflammation TLO can arise (13), and this likely applies to LV-GCA patients where diagnosis can be delayed or missed due to non-specific symptoms. In C-GCA, diagnosis is set sooner and treatment starts immediately to prevent blindness. In the positive TAB of one patient, we did observe a degree of B/T cell co-localization which is the first step toward ATLO formation. This specific patient complaint of persistent fatigue after cessation of glucocorticosteroid treatment and aortic aneurysm surgery was performed 5 years later. In the aorta tissue of this specific patients we also observed extensive B-cell infiltration and ATLO formation suggesting that disease duration could indeed be important. B-cells and ATLOs could be a target for imaging in these difficult to diagnose LV-GCA patients.

The major strength of our study is the comprehensive immunohistochemistry analysis in an unique cohort of LV-GCA patients compared with age and sex-matched atherosclerosis patients. This study is limited by the small number of aortic tissue studied. LV-GCA patients rarely undergo aortic surgery which limits the amount of tissue available for research. Another limitation is the retrospective nature of the study, with limited clinical data and no blood biobanking of these patients being available before aortic surgery.

In conclusion, aortas of LV-GCA patients show massive infiltration of B-cells that outnumber the T-cells and can organize into ATLOs at the adventitial site, with germinal centers, proliferating B-cells, and plasma cell niches. Our data strongly suggest a role for B-cells in the pathogenesis of GCA and thus urge further investigation into the factors determining the tissue tropism, organization and function of B-cells in different stages of GCA.

## Data Availability Statement

The raw data supporting the conclusions of this manuscript will be made available by the authors, without undue reservation, to any qualified researcher.

## Author Contributions

JG, AB, EH, AD, EB, and MS: study design and acquisition, analysis, and interpretation of data; JG, AB, EB, and MS: manuscript preparation; AB, EB, and MS: overall supervision. All authors read and commented on the manuscript.

### Conflict of Interest Statement

The authors declare that the research was conducted in the absence of any commercial or financial relationships that could be construed as a potential conflict of interest.
