# Supplementary material for: Massive B-Cell Infiltration and Organization Into Artery Tertiary Lymphoid Organs in the Aorta of Large Vessel Giant Cell Arteritis
Source: Front Immunol. 2019 Jan 29;10:83. doi: 10.3389/fimmu.2019.00083 (PMC6361817; doi:10.3389/fimmu.2019.00083)
Supplement: Supplementary file 1 [file Table_1.DOCX]

Supplementary Material

Massive B-cell infiltration and Organization into artery tertiary lymphoid organs in the aorta of Large Vessel Giant Cell Arteritis

Jacoba C. Graver*, Annemieke M.H. Boots, Erlin A. Haacke, Arjan Diepstra, Elisabeth

Brouwer, Maria Sandovici

*** Correspondence:** Jacoba C. Graver: j.c.graver@umcg.nl

# Supplementary Table

| **Antibody** | **Isotype** | **Clone** | **Supplier** | **Dilution** | **Antigen retrieval** |
| --- | --- | --- | --- | --- | --- |
| CD20 | Mouse IgG2a | L26 | Ventana Medical Systems | ready to use | 1mM EDTA (pH=8) |
| CD3 | Rabbit IgG | 2GV6 | Ventana Medical Systems | Ready to use | 1mM EDTA (pH=8) |
| CD21 | Mouse IgG2a | 2G9 | Ventana Medical Systems | Ready to use | 1mM EDTA (pH=8) |
| Bcl6 | Mouse IgG1 | GI191E/A8 | Ventana Medical Systems | Ready to use | Ultra CC1 36 minutes |
| PNAd | Rat IgMκ | Meca-79 | Novus Biologicals | 1:200 | 10MM citrate (pH=6) |
| CD68 | Mouse IgG3κ | PG-M1 | DAKO | 1:50 | 10mM tris-HCL+1mM EDTA (pH=9) |
| CD138 | Mouse IgG1κ | MI15 | DAKO | 1:50 | 10mM tris-HCL+1mM EDTA (pH=9) |
| Ki-67 | Mouse IgG1κ | MIB-1 | DAKO | 1:50 | 10mM tris-HCL+1mM EDTA (pH=9) |
| Adipophilin | Rabbit IgG | EPR3713 | Abcam | 1:200 | Ultra CC1  64 minutes |
| Anti-IgM | Rabbit | Polyclonal | Ventana Medical Systems | Ready to use | Ultra CC1 20 minutes |
| IgG | Rabbit | polyclonal | Ventana Medical Systems | Ready to use | Ultra CC1 20 minutes |
| IgG4 | Mouse IgG1κ | MRQ-44 | Roche | Ready to use | Protease 1 8 minutes |
| **Table S1.** Primary antibodies and antigen retrieval used for immunohistochemistry | | | | | |
